# Tongue acupuncture for the treatment of post-stroke dysphagia: a meta-analysis of randomized controlled trials

**DOI:** 10.3389/fnins.2023.1124064

**Published:** 2023-05-25

**Authors:** Li Li, Fei Xu, Shengping Yang, Peng Kuang, Haoying Ding, Mei Huang, Chunyan Guo, Zishui Yuan, Xiao Xiao, Zuhong Wang, Pengyue Zhang

**Affiliations:** ^1^Department of Acupuncture, Kunming Municipal Hospital of Traditional Chinese Medicine, Kunming, China; ^2^Key Laboratory of Acupuncture and Massage for Treatment of Encephalopathy, College of Acupuncture, Tuina and Rehabilitation, Yunnan University of Traditional Chinese Medicine, Kunming, China

**Keywords:** tongue acupuncture, acupuncture therapy, apoplexy, dysphagia after stroke, systematic review, meta analysis

## Abstract

**Objectives:**

Post-stroke dysphagia is the most common neurological impairment after stroke. The swallowing process is controlled by a network made up of the cerebral cortex, subcortical area, and brainstem structure. The disruption of the swallowing network after stroke leads to dysphagia. The affected swallowing muscles after stroke mainly include the laryngeal muscles (suprahyoid muscle and thyrohyoid muscle) and infrahyoid muscle. These muscles experience kinematic effects and muscle strength weakens, resulting in reduced movement in the swallowing process. Acupuncture can change the excitability of cerebral cortical nerve cells, promote the recovery of neurological function, and enhance neuromuscular excitability, ultimately improving the control of swallowing-related nerves and muscles and promoting swallowing functional recovery. In this meta-analysis, we systematically evaluate the clinical efficacy of acupuncture in the treatment of post-stroke dysphagia.

**Methods:**

Randomized controlled trials of tongue acupuncture therapy for post-stroke dysphagia were searched and selected from seven electronic databases (PubMed, CBM, Cochrane, Embase, CNKI, VPCS, and Wan fang). The Cochrane Collaboration tool was used to conduct methodological quality assessment. Rev. Man 5.4 software was utilized to perform data analysis.

**Results:**

A total of 15 studies with 1,094 patients were included. Meta-analysis Showed that WST score WST score (MD = −0.56, 95% CI (−1.23, 0.12), Z = 1.62, *p* < 0.00001), SSA score (MD = −1.65, 95% CI (−2.02, −1.28), Z = 8.77, *p* < 0.00001). These results suggested that the treatment group (tongue acupuncture or tongue acupuncture combined with other therapies) was superior to the control group in reducing WST scores and SSA scores. The clinical efficacy of the tongue acupuncture group was better compared with the control group (MD = 3.83, 95% CI (2.61, 5.62), Z = 6.88, *p* < 0.00001).

**Conclusion:**

The meta-analysis showed that the total effective rate of patients with dysphagia after stroke in the treatment group (acupuncture, tongue acupuncture, and acupuncture combined with other therapy) was higher than that in the control group. These results indicated that acupuncture, tongue acupuncture, and acupuncture combined with other therapy can improve post-stroke dysphagia.

## 1. Introduction

Stroke is an acute cerebrovascular disease defined as ischemia or hemorrhage, of which ischemic stroke accounts for 76% (Virani et al., 2021), and leads to a variety of neurological defects. Statistics show that there are 2.4 million new stroke patients in China every year, about 1.1 million deaths, and 11 million stroke patients, most of which are ischemic stroke, and these statistics show an upward and younger trend year by year. China has become the country with the heaviest burden of stroke in the world (Zhou et al., 2019). Data show that 29–78% of stroke patients have dysphagia (Qiao et al., 2022), and the mean incidence of the disease is 50% (Du et al., 2021). 91% of patients with post-stroke dysphagia are mild (Umay et al., 2013; Cohen et al., 2016). Although the survival rate of stroke patients has been significantly improved because of the improvement of medical skills such as first aid and thrombolysis, most survivors of stroke are affected by sequelae such as dysphagia and speech, motor, and memory impairments (Barthels and Das, 2020). Stroke is the most common neurological cause of dysphagia (Cui et al., 2020). Dysphagia is a process in which food cannot be transported safely and efficiently to the stomach because of the damaged structure and function of organs, such as the jaw, the Soft Palate, the lips, the tongue, the throat, the esophagus, etc. Swallowing muscles mainly include the laryngeal muscles (suprahyoid muscle and thyrohyoid muscle) and the subglottis. These muscles experience kinematic effects and muscle strength weakens, resulting in reduced movement in the swallowing process (Jung et al., 2020). Swallowing is one of the most complex somatic reflexes. It is controlled by the cerebral cortex, cortical medulla oblongata pathway, brainstem, the swallowing center, and pairs 3rd, 4th, 5th, 6th, and 7th of the cerebral nerves and C1, C2, and C3 of the spinal nerves (Jean, 2001; Michou and Hamdy, 2009). Stroke disrupts the swallowing network and can lead to dysphagia. According to the location of food passing through, swallowing can be divided into four stages: pre-oral stage, oral stage, pharyngeal stage, and esophageal stage (Chinese Expert Consensus Group for Rehabilitation Evaluation and Treatment of Dysphagia, 2017). The majority of post-stroke dysphagia occurs in the delivery of food and fluid from the oral cavity to the stomach, and their dysfunction occurs primarily in the oral and pharyngeal phases. It often manifests as saliva or food coming out of the mouth, holding food in the mouth for a long time without swallowing, food or water coming out of the nose (nasal reflux), food sticking to the mouth or throat, and bucking when eating or drinking. Moreover, dysphagia can lead to bucking, aspiration pneumonia, malnutrition, etc. Severe cases endanger life due to asphyxia. Thus, post-stroke dysphagia seriously affects patients’ quality of life and increases family and social burdens, and it is necessary to find an effective strategy for promoting the functional recovery of patients with post-stroke dysphagia.

The European Stroke Organization and the European Society for Dysphagia have jointly developed the 2021 European guidelines for the diagnosis and treatment of dysphagia after Stroke (Dziewas et al., 2021). The guidelines recommended that the treatment for post-stroke dysphagia include dietary interventions, nutritional interventions, behavioral interventions (swallowing training), oral health, medication (Capsaicin receptor 1 agonist and dopaminergic drugs), and peripheral or central nervous regulation (repetitive trans cranial magnetic stimulation, trans cranial electrical stimulation, trans cranial direct current stimulation, and pharyngeal electrical stimulation) (Ye et al., 2022). However, so far there is no specific and effective therapeutic schedule for the treatment of post-stroke dysphagia. In China and some East Asian countries, acupuncture has been widely used in the treatment of stroke and achieved a good curative effect. Tongue acupuncture is a special micro-acupuncture therapy. Clinical practice has proved that tongue acupuncture is an effective treatment for dysphagia after a stroke. Tongue acupuncture is a kind of swift pricking blood therapy; the acupuncture therapy has the benefits of being fast, with little pain and no side effects. It is easy to administer by acupuncturists and well-accepted by patients.

In traditional Chinese medicine, post-stroke dysphagia can be classified into the “she jian” (which means sluggish tongue impeding speech) and “yin fei (which means the tongue is paralyzed and cannot work well). Their main clinical manifestations are slow rotation of tongue, uncontrolled eating, and loss of speech. Acupuncture is an effective and internationally recognized treatment of stroke that can significantly reduce the disability rate. Acupuncture can stimulate nerve terminal receptors, help nerve sensory input, promote the recovery of the damaged cerebral cortex and subcortical nerve, improve the function of the glossopharyngeal nerves and the reflex arc, and enhance the swallowing reflex (Guan et al., 2016; Zhang, 2017).

Tongue acupuncture is a special micro-acupuncture therapy created by famous acupuncturist Guan Zhengzhai, based on the theory from Huang Di Nei Jing of the relationship between tongue and Zangfu-meridians theoretic and modern biological holography, combined with decades of clinical experience, and has become an important part of acupuncture together with ear and head acupuncture methods (Guan et al., 2021).

According to the theory of Chinese medicine, the heart may be reflected on the tongue, which is connected directly or indirectly with the Zangfu-meridians theoretic by the circulation of the meridians and the infusion of qi and blood, closely connected with the heart, spleen, and kidneys. The heart is said to govern blood and vessels as well as the spirit. The tongue is governed by the heart-mind and brain marrow. Stimulation of the tongue may promote brain function repair and improve post-stroke dysfunction through “blood-vessel-heart-spirit.” Acupuncture points on the tongue stimulate the connected meridians or Zangfu-meridians theoretic in order to regulate qi and blood flow, opening and closing the orifices, and at the same time nourishing the blood channels of the tongue, smoothing the tongue meridians, and promoting tongue and pharyngeal recovery. Clinical practice shows that tongue acupuncture therapy is less painful for the patient, is easy to administer, and is more effective when combined with body acupuncture. Tongue acupuncture in the treatment of dysphagia can improve swallowing function by changing the excitability of cortical nerve cells, promoting the recovery of neurological function, enhancing neuron muscular excitability, activating related pathways or the combination of both, and improving the control of swallowing-related nerves and muscles. Thus, tongue acupuncture is an effective, safe, and reliable traditional Chinese medicine therapy with many years of clinical experience and is a potential method for the treatment of dysphagia.

This article aimed to evaluate the effectiveness of tongue acupuncture in the treatment of post-stroke dysphagia by Meta-analysis, and we hope to provide a reliable therapy for the treatment of post-stroke dysphagia and to promote its clinical application.

## 2. Methods

### 2.1. Search strategy

We aggregated all the data about tongue acupuncture treatment dysphagia after stroke from the Cochrane Library, PubMed, Embase, China Biomedical Literature Service (Sino Med), Chinese journal full-text database (CNKI), wan fang databases, Chinese Science and Technology Journal Database (VPCS), and China Biomedical Literature Database (CBM). The data aggregation time is from the establishment of the database to the present. Chinese search terms included “tongue acupuncture,” “tongue triple acupuncture,” “acupuncture,” “stroke,” “pseudo bulbar palsy,” “cerebral stroke,” “swallowing disorder,” “systematic evaluation,” “randomized controlled,” “clinical study,” “clinical observation,” and “Meta-analysis.” The English search terms included “acupuncture,” “tongue acupuncture,” “stroke,” “pseudo bulbar paralysis,” “appetite disorder,” “dysphagia,” “systematic review,” “RCT,” “clinical study,” and “meta-analysis.”

### 2.2. Eligibility criteria

#### 2.2.1. Inclusion criteria

The inclusion criteria were As follows:

1. Study subjects met diagnostic criteria for post-stroke dysphagia.
2. The intervention method involves tongue acupuncture.
3. Published randomized controlled trials (RCT).
4. The main efficiency measurements are the sub-water test and clinical efficacy; the secondary efficiency measurement is the SSA score and VFSS score.
5. The study protocol is reasonably designed, with clear proposals for acupuncture operations, treatment procedures, etc.

#### 2.2.2. Exclusion criteria

The exclusion criteria were As follows:

1. Repeated studies in the literature, with one article retained.
2. Clinical case reports, review articles, animal studies, conference papers, papers that do not involve control groups, and multiple studies.
3. The research design is unreasonable (such as intervention measures, random methods, etc.)
4. Studies with unclear diagnostic criteria and criteria for determining efficacy.

### 2.3. Literature screening

We imported the obtained literature data into Note Express software, and then the duplicate literature were removed through the automatic review and manual review function. Two reviewers (LL and KP) independently reviewed the titles and abstracts of the studies according to the eligibility criteria. If potential studies met the criteria, further full-text evaluation was required. Studies that remained controversial would be arbitrated by a third researcher (XF). We used Note Express software (Version 3.7) to manage the retrieved records.

### 2.4. Data extraction

Two reviewers (LL and KP) extracted the following data independently of each other: first author, year of publication, simple intervention measures, tongue acupoints, treatment time, and results criteria; the extracted data were then cross-checked.

### 2.5. Quality evaluation

We carefully read the literature, strictly followed the inclusion and exclusion criteria, extracted the data, and referred those elements that generated disagreement and uncertainty during the screening process to a third evaluator for the final decision. The Cochrane Risk of Bias tool was used to achieve a methodological quality assessment of the literature (Higgins Julian and Sally, 2008). There were six main components: methods of random allocation, concealment of allocation scheme, blind methods, completeness of outcome data, selective reporting, other sources of bias, and the achievement of risk judgments after subjecting their content to certainty.

### 2.6. Criteria for clinical efficacy

The evaluation standard of clinical curative effect was drawn up according to the improvement of clinical symptoms of patients and the results of the Kubota water drinking test:

1. Cured: The swallowing function returned to normal, the clinical symptoms disappeared, and the Kubota water drinking test reached grade 1.
2. Significantly effective: The swallowing function basically returned to normal, the symptoms basically disappeared, and the Kubota water drinking test reached level 2.
3. Effective: The swallowing function was improved, and the Kubota water drinking test improved from grade 4 or 5 to grade 3 after treatment.
4. Ineffective: After treatment, the patient’s swallowing dysfunction did not improve, and the Kubota water drinking test did not improve, or even worsened.

### 2.7. Statistical analysis

Statistical analysis was performed by Rev. Man 5.4 software. Using C^2^ and I^2^ tests to assess data heterogeneity, the random effects model was selected when statistical heterogeneity was significant (*p* < 0.10 or I^2^ ≥ 50%). When statistical heterogeneity was not significant (*p* ≥ 0.10 or I^2^ ≤ 50%), the fixed effects model was selected. The odds ratio (OR) and 95% confidence interval (CI) for dichotomous variables were used to express the statistics of the efficacy analyses. For continuous data, weighted mean differences (MD) and 95% confidence intervals (CI) were used to express efficacy analysis statistics. The potential publication bias was analyzed by using an “inverted funnel” diagram, and bias in included trials was discussed.

## 3. Results

### 3.1. Basic characteristics of search results and included literature

**Figure 1:**
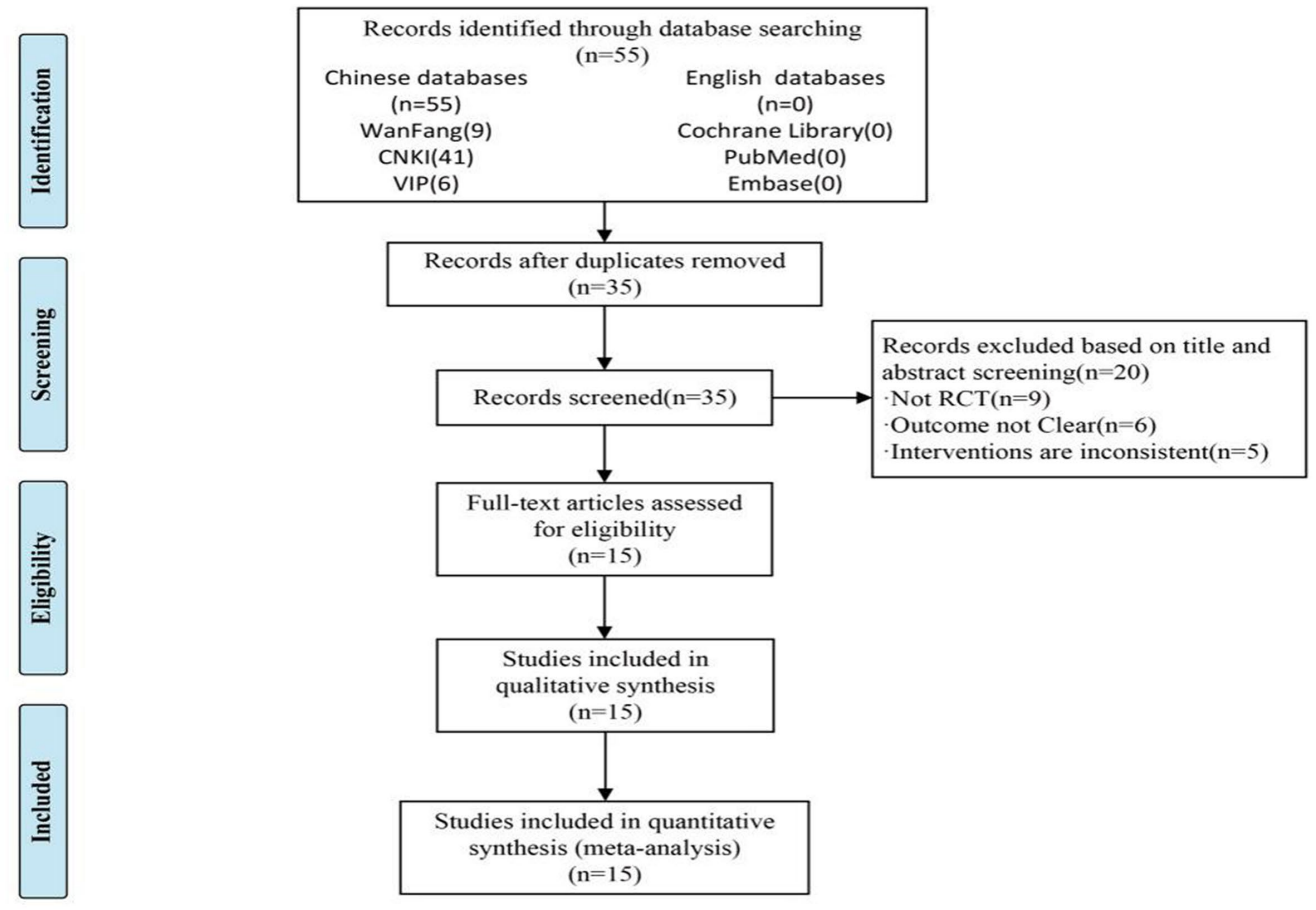
Flow chart of study selection process.

Through the database, 55 articles were searched. After importing them to Note Express and removing duplicates, 31 articles remained. By reading the title and abstract, and strictly applying the inclusion and exclusion criteria, 20 papers remained. After detailed reading of the full texts of each article, 15 RCTs were finally selected (Jean, 2001; Li et al., 2005; Higgins Julian and Sally, 2008; Shuai and Jianqiao, 2008; Liu et al., 2009; Michou and Hamdy, 2009; Wei, 2012; Li et al., 2013; Guan et al., 2016; Chinese Expert Consensus Group for Rehabilitation Evaluation and Treatment of Dysphagia, 2017; Zhang, 2017; Jung et al., 2020; Dziewas et al., 2021; Guan et al., 2021; Ye et al., 2022), for a total of 1,094 patients. Figure 1 shows a flow diagram of the study. The basic characteristics of the included research literature are shown in Table 1.

**Table 1:** Basic features of the included literature.

Study	Number	Intervention measures		Age (y)		Gender (M/F)		Days	Results
	T/C	T	C	T/C		T/C			Criteria
Li et al. (2005)	32/30	Tongue acupuncture	body acupuncture	60.5 ± 5.3	61.8 ± 6.2	20/12	18/12	24 days	③⑥⑦
Shuai and Jianqiao (2008)	39/39	Tongue acupuncture + body acupuncture	body acupuncture	68.81 ± 3.57	68.89 ± 4.11	23/16	21/18	34 days	①⑥
Liu et al. (2009)	100/100	Nape acupuncture +head acupuncture+ Tongue acupuncture+Swallowing training	Swallowing training	NA	NA	NA	NA	42 days	①⑥
Wei (2012)	31/30	Glossopharyngeal acupuncture	common acupuncture	66.8	67.2	18/13	16/14	28 days	①⑥
Li et al. (2013)	30/30	control group based +Tongue acupuncture+Electrical stimulation	Western medicine + Rehabilitation training	58.32	59.68	18/12	18/12	45 days	①⑥
Wei (2012)	40/40	Nape acupuncture +Tongue acupuncture	Nape acupuncture	63.2	61.9	26/14	25/15	28 days	①⑥
Gao et al. (2014)	52/49	Tongue acupuncture +Dysphagia therapeutic apparatus	Dysphagia therapeutic apparatus	60.25 ± 8.36	61.37 ± 7.36	31/21	27/22	29 days	①⑥⑦
Shen et al. (2015)	60/60	Tongue acupuncture	Feng chi、nei guan	NA	NA	NA	NA	28 days	④⑥
Yang and Chen (2017)	24/24	Tongue acupuncture+Catheter balloon dilatation	Catheter balloon dilatation	60 ± 8	60 ± 5	14/10	13/11	10 days	①
Liu et al. (2018)	30/30	Tongue acupuncture	Nape acupuncture	59.2 ± 6.92	59.43 ± 6.7	22/8	24/6	28 days	①
Wang and Shen (2019)	30/30	Swallowing training + head acupuncture+Tongue acupuncture	Swallowing training	55.86 ± 8.93	56.12 ± 9.04	21/9	17/13	42 days	①⑥
Shao (2019)	30/30	Rehabilitation training+head acupuncture+Tongue acupuncture	Rehabilitation training	58.45 ± 2.36	21.13 ± 2.28	18/12	17/13	28 days	①⑥
Xiao et al. (2020)	36/36	Tongue acupuncture+The seven points of the skull base acupuncture	Common acupuncture	57.11 ± 9.37	56.39 ± 10.84	22/14	25/11	14 days	①⑥
Cui et al. (2021)	40/40	Swallowing training+Tongue acupuncture	Swallowing training	NA	NA	NA	NA	14 days	⑥
Liu and Wan (2021)	30/30	Common acupuncture Tongue acupuncture +Combined exercise imagination therapy	Common acupuncture	58.36 ± 4.32	52.57 ± 3.58	17/13	16/14	28 days	①⑥

①WST score, ②SPECT, ③SSA, ④VFSS, ⑤s EMG, ⑥Total efficiency, ⑦Dysphagia score, ⑧SWAL-QOL, ⑨NIHSS.

### 3.2. Risk of bias

**Figure 2:**
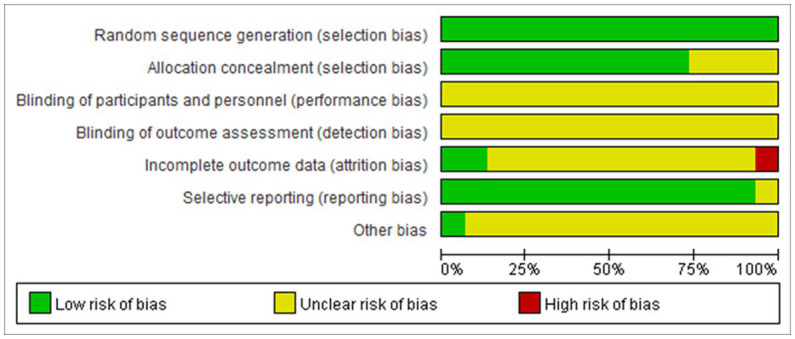
Risk of bias percentile bar graph.

**Figure 3:**
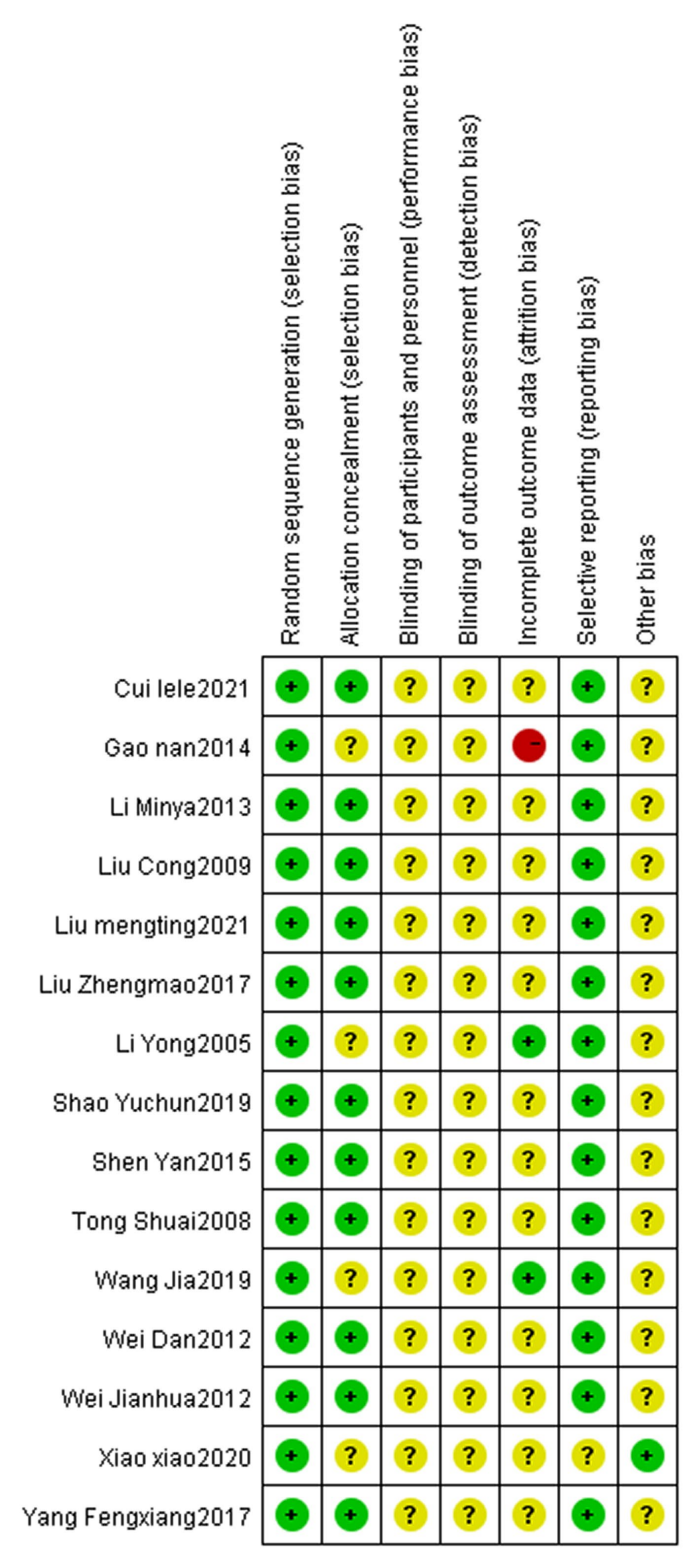
Risk of bias summary graph.

There are relatively few clinical reports on the treatment of dysphagia after apoplexy with tongue acupuncture. The included articles have complete data without selective reporting or other bias. However, only four (Gao et al., 2014; Wang and Shen, 2019; Xiao et al., 2020; Liu and Wan, 2021) describe random methods in detail, and none of them mention allocation hiding. No follow-up data were reported for all outcome data, and no reason for loss of follow-up was mentioned. This is shown in Figures 2, 3.

## 4. Results of the meta-analysis

### 4.1. Clinical efficacy

**Figure 4:**
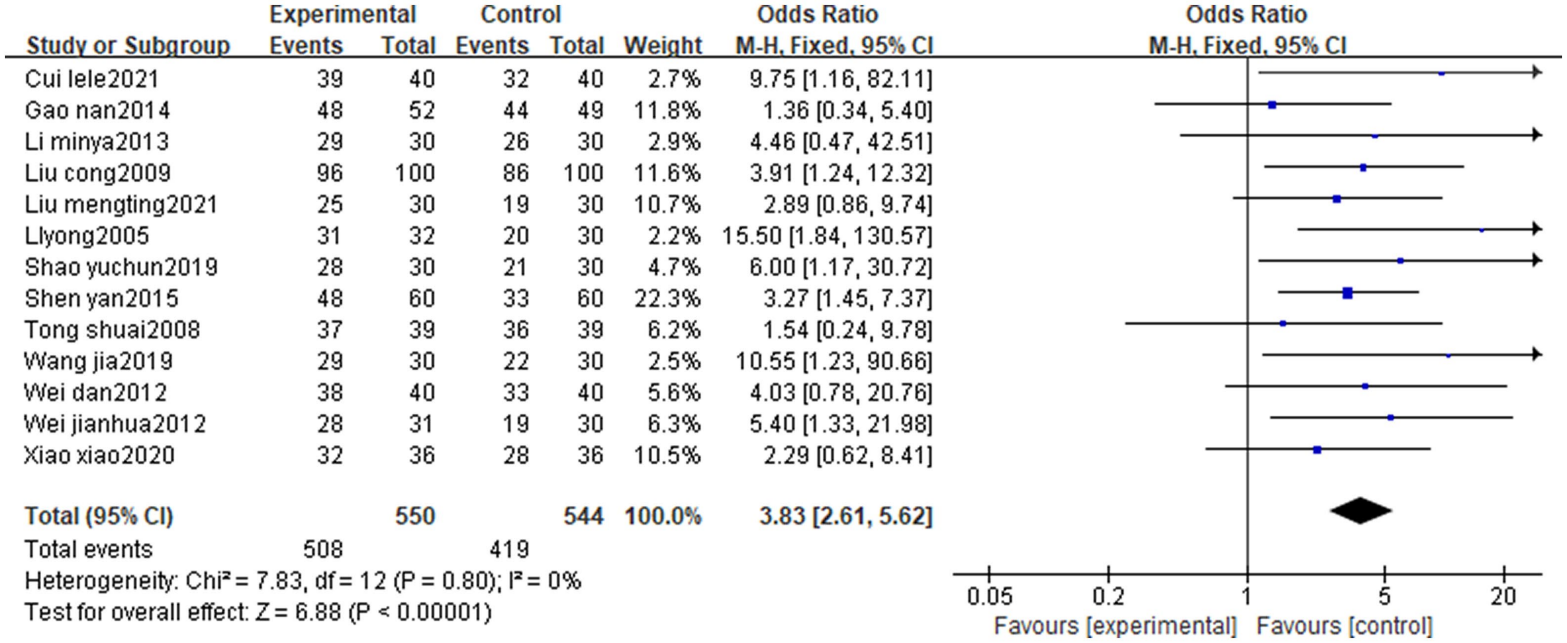
Total effective rate of tongue acupuncture for post-stroke dysphagia.

A total of 15 cases were included in the literature, and 13 studies (Li et al., 2005, 2013; Shuai and Jianqiao, 2008; Liu et al., 2009; Wei, 2012; Gao et al., 2014; Shen et al., 2015; Shao, 2019; Wang and Shen, 2019; Xiao et al., 2020; Cui et al., 2021; Liu and Wan, 2021) evaluated the efficiency rate with good homogeneity (*p* = 0.8, I^2^ = 0%) and statistically significant differences [MD = 3.83, 95% CI (2.61, 5.62), Z = 6.88, *p* < 0.00001]. It was suggested that the overall efficiency of the treatment group (tongue acupuncture or tongue acupuncture combined with other therapies) for patients with post-stroke dysphagia was higher than that of the control group. See Figure 4 for details.

### 4.2. WST score

**Figure 5:**
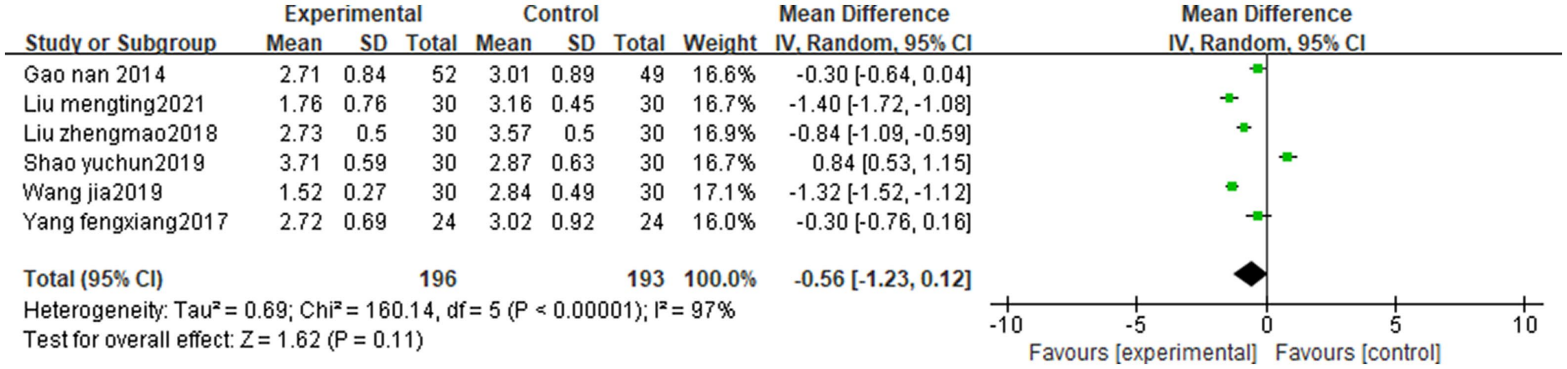
Sub-water test of tongue acupuncture for post-stroke dysphagia.

In the literature included in this study, a total of 11 articles used the sub-water test as an efficacy criterion, two articles observed the grading criteria of the sub-water test, three articles did not statistically analyze the data, and six studies (Gao et al., 2014; Yang and Chen, 2017; Liu et al., 2018; Shao, 2019; Wang and Shen, 2019; Liu and Wan, 2021) used the sub-water test score and met the conditions of meta-analysis. Inter-study heterogeneity was large (*p* < 0.00001, I^2^ = 97%). Subgroup analysis showed that the WST scores in the treatment group (tongue acupuncture or tongue acupuncture combined with other therapies) were better than the control group [MD = −0.56, 95% CI (−1.23, 0.12), Z = 1.62, *p* < 0.00001]. See Figure 5 for details.

### 4.3. SSA score

**Figure 6:**
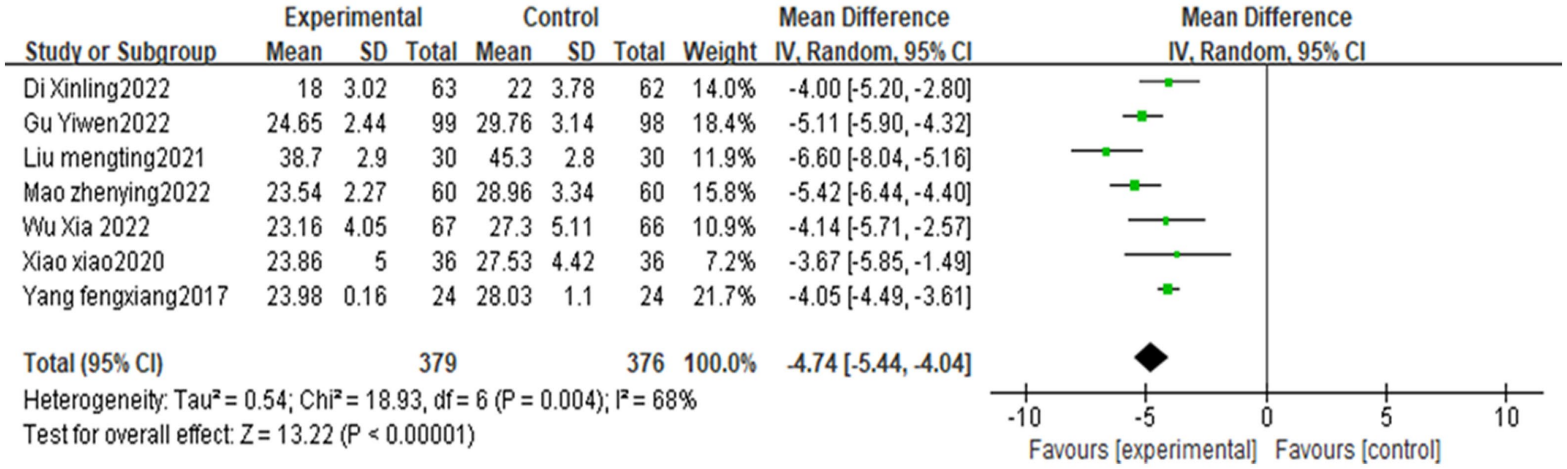
Meta-analysis of the swallowing function assessment scale after stroke treated with acupuncture.

In the literature included in this study, a total of three studies (Yang and Chen, 2017; Xiao et al., 2020; Liu and Wan, 2021) used the Swallowing Function Assessment Scale scores, with large inter-study heterogeneity (*p* < 0.00001, I^2^ = 96%), and differences between studies were statistically significant. Subgroup analysis showed that tongue acupuncture or tongue acupuncture combined with other therapies significantly improved the SSA score compared with the control group [MD = −1.65, 95% CI (−2.02, −1.28), Z = 8.77, *p* < 0.00001]. See Figure 6 for details.

### 4.4. Evaluation of publication bias

In this study, the funnel plot method was used to evaluate the publication bias of the main outcome indicators, sub-water test scores, and clinical efficacy.

#### 4.4.1. Publication bias of clinical efficacy

**Figure 7:**
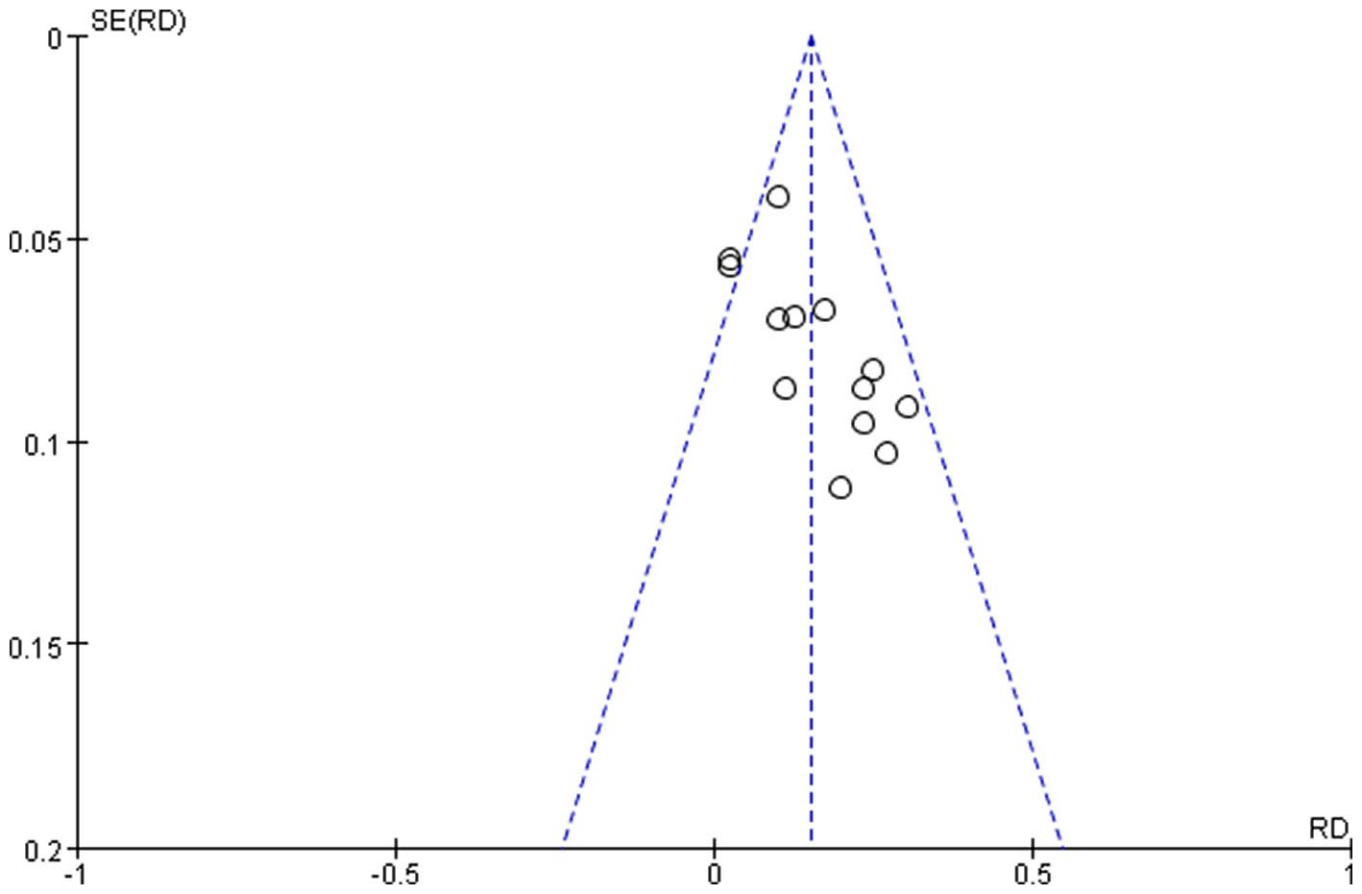
Publication bias of clinical efficacy.

The clinical efficacy of the 13 included studies [19, 20, 21, 22, 23, 24, 25, 26, 29, 30, 31, 32, 33] was evaluated for publication bias, and the funnel plot pattern was approximately symmetrical to the left and right, which indicated that publication bias may not exist. See Figure 7 for details.

## 5. Discussion

According to the statistical data from the American Heart Association, stroke has become the second leading cause of death in the world, characterized by high rates of morbidity, mortality, disability, and recurrence. Health education guidelines emphasize that rational prevention and timely treatment are indispensable components of the clinical diagnosis and treatment strategy for stroke. Dysphagia is one of the accompanying symptoms of stroke patients. Acupuncture can effectively ameliorate swallowing impairments after a stroke. Acupuncture can increase the sensorimotor stimuli of these nerve-controlling muscles, and these repetitive stimuli can help to activate the central nervous system related to swallowing and enhance neuromuscular excitability. In China, acupuncture is more commonly used in the treatment of post-stroke swallowing disorders. Acupuncture can promote the pharynx, replenishing qi and dredging collateral. The stimuli of the acupuncture points on the tongue can unblock the meridians, qi, and blood, improve the motor function of the tongue, improve the pharyngeal nerve response, promote the construction of the swallowing reflex arc, restore the regulation of the cortical brain bundle, and improve the swallowing function and the quality of life. The randomized controlled studies’ literature included in this study were related to tongue acupuncture for the treatment of post-stroke dysphagia. The clinical observation shows that tongue acupuncture can improve dysphagia after stroke, which has certain clinical significance. The aim of this meta-analysis was to provide high-quality evidence-based medical evidence for the clinical treatment of stroke dysphagia.

There are some limitations to this study: (1) All included articles were reported in China, and no relevant clinical reports were found in foreign language databases, which may affect the promotion and application of tongue acupuncture in foreign countries; (2) The quality of included articles was relatively low, and no relevant clinical reports were found. Concealment of configuration and partial mention of blinding methods may affect the credibility of the results; (3) No study reported follow-up data, and none mentioned the reasons for loss of follow-up, so it is difficult to predict long-term efficacy; and (4) Clinical studies of post-stroke dysphagia were mostly observed using subjective indicators, and while SSA is an internationally recognized tool for the assessment of dysphagia, WST is the most classical and concise screening method for dysphagia with the advantages of easy accessibility and patient tolerance. VFSS and FEES are the gold standards for the diagnosis of dysphagia. Electromyography can record the muscle activity of superior and inferior hyoid muscles (Zhang et al., 2022). According to the literature (Yang, 2019; Di et al., 2022; Gu et al., 2022; Wu et al., 2022), VFSS and EMG were used as observation indexes to evaluate the curative effect. However, the stability of the results is not satisfactory, and this may be related to the combination of acupuncture of the tongue with other therapies and the intensity of the acupuncture treatment. More objective indicators should be used in the future, and further studies are still needed for further analysis and validation.

This study still has some limitations. According to the Cochrane Risk of Bias tool, which uses randomization, allocation concealment, blind assessment, none of them mentioned assigning hidden. Due to the inclusion of relatively small sample sizes with a maximum of 100 cases and a minimum sample size of 24 cases, and the deficiency of an estimate of sample size, with experimental protocols not rigorously designed and baseline treatments not explicitly mentioned, this may have led to some heterogeneity.

## 6. Conclusion

The meta-analysis indicated that tongue acupuncture or tongue acupuncture combined with other therapies is clinically effective in the treatment of post-stroke dysphagia. However, there were some shortcomings in the literature included in this study. Because the evaluation of the methodological quality and quality of evidence is a subjective process and different researchers make independent judgments on each factor, the results of the studies may vary somewhat. In addition, acupuncture is difficult for the blind method in its implementation, and most experimental protocols are single-blind. In the future, randomized clinical studies with high quality, multi-center, large samples, and regular follow-up should be further carried out to improve the research quality. Strict design of the experimental scheme and adopting scientific research methods are also crucial to provide more meaningful evidence for the clinic.

## Author contributions

LL, PZ, and ZW revised the manuscript. LL and FX identified studies and conducted data collection, extraction, and analyzed all the data. PK performed validation of data. LL completed the first draft. PZ provided guidelines for this meta-analysis. All authors contributed to the article and approved the submitted version.

## Funding

This study was supported by the Yunnan Acupuncture Clinical Research Center (2022-09-01-015), National Natural Science Foundation of China (81860881), General Project of Applied Basic Research Program of Yunnan Province (2019FB118), and Yunnan Science and Technology Department Joint Special Fund Project (2017FF116-041, 2018FF001-016, and 2018FF001-079).

## Conflict of interest

The authors declare that the research was conducted in the absence of any commercial or financial relationships that could be construed as a potential conflict of interest.

## Publisher’s note

All claims expressed in this article are solely those of the authors and do not necessarily represent those of their affiliated organizations, or those of the publisher, the editors and the reviewers. Any product that may be evaluated in this article, or claim that may be made by its manufacturer, is not guaranteed or endorsed by the publisher.
